# Towards effective and sustainable global academic partnerships through a maturity model informed by the capability approach

**DOI:** 10.1186/s12992-021-00785-2

**Published:** 2021-11-20

**Authors:** Abebaw Fekadu, Esubalew Assefa, Abraham Tesfaye, Charlotte Hanlon, Belete Adefris, Tsegahun Manyazewal, Melanie J. Newport, Gail Davey

**Affiliations:** 1 Centre for Innovative Drug Development and Therapeutic Trials for Africa (CDT-Africa), Addis Ababa University, Addis Ababa, Ethiopia; 2 Global Health & Infection Department, Brighton and Sussex Medical School, Brighton, UK; 3 Department of Psychiatry, WHO Collaborating Centre for Mental Health Research and Capacity-Building, School of Medicine, College of Health Sciences, Addis Ababa University, Addis Ababa, Ethiopia; 4 Department of Economics, College of Business and Economics, Jimma University, Jimma, Ethiopia; 5 Health Services and Population Research Department, Centre for Global Mental Health, King’s College London, London, UK; 6 School of Public Health, College of Health Sciences, Addis Ababa University, Addis Ababa, Ethiopia

**Keywords:** Academic partnership maturity model, Global partnerships, Sustainable development goals, Capability

## Abstract

**Background:**

Shortage of skilled workforce is a global concern but represents a critical bottleneck to Africa’s development. While global academic partnerships have the potential to help tackle this development bottleneck, they are criticised for inadequate attention to equity, impact, and sustainability. We propose a new values-driven partnership model for sustainable and equitable global partnerships that achieve impact.

**Method:**

The model was based on the authors’ experiences of participation in over 30 partnerships and used insights from the Capability Approach.

**Results:**

We developed an Academic Partnership Maturity Model, with five levels of maturity, extending from pre-contemplative to mature partnerships. The level of maturity increases depending on the level of freedom, equity, diversity, and agency afforded to the partners. The approach offers a framework for establishing a forward-looking partnership anchored in mutual learning, empowerment, and autonomy.

**Conclusion:**

This is a pragmatic model limited by the biases of experiential knowledge. Further development of the concept, including metrics and an evaluation tool kit are needed to assist partners and funders.

## Background

The potential of global partnerships to assist low- and middle-income countries as they strive to address their development challenges is substantial [1, 2]. This is particularly relevant when building skilled workforce through academic partnerships. While shortage of skilled workforce is a global concern [3], it represents a serious threat to Africa’s development [3, 4] and impedes Africa’s potential to contribute to global welfare. For example, whereas globally, over 1000 researchers are available per million population, only 92 are available in Africa [5]. In the context of population growth, climate change and pandemics, this severe shortfall in expertise can translate into an existential threat for Africa. Global partnerships may be a major path to rectifying this gap. In recognition of its potential role, global partnerships have taken a central role in the global development agenda expressed in the Millennium Development Goals (MDG) and the Sustainable Development Goals (SDG). The last goal (Goal 17) of the SDGs is dedicated to partnership as a means of achieving the SDGs, with at least eight of the 19 targets referring to partnership and capacity building. These targets emphasise cooperation for knowledge sharing, technology transfer and innovation to support national plans for achieving sustainable development [2].

Alongside the SDGs, the United Nations Industrial Development Organization (UNIDO) has proposed a Programme for Country Partnership (PCP) [6] aimed at “accelerating inclusive and sustainable industrial development”. The PCP encourages mobilisation of “partners and resources to achieve larger development impact”. A recent World Bank Africa Centres of Excellence initiative, which recognises the lack of skilled manpower in the sciences as a critical development challenge for Africa, employs partnership as a major tool to support African higher education institutions to address this critical challenge [4]. Another important initiative, the Partnership for Applied Science, Engineering, and Technology, is designed to leverage a partnership with countries in Asia and Latin America (Brazil, China, India and the Republic of South Korea) to improve knowledge and expertise in the applied sciences [5].

However, although undoubtedly important, global academic partnerships are complex [7], influenced by relationship dynamics, geopolitical issues and mismanagement. They are often short-term, with an agenda that is not always co-developed. Furthermore, at present, the focus of global partnerships is primarily on addressing system or process challenges rather than on functions that are more likely to lead to autonomy, freedom, and mutual benefit, for example, development of critical technological expertise and infrastructure. Achieving sustainable development through partnership requires longer-term and predictable engagement between partners guided by considerations of mutual growth, maturity and sustainability. The various guidelines and principles put forward to ensure sustainability and equity [8–11] do not offer guidance on how partnerships should be built to last. The main objective of this commentary is to propose a value driven maturity model for academic partnerships informed by the Capability Approach [12, 13]. This model may assist academic relationships to develop strategically with a clear intent for longer-term impactful engagements. We first expand on the rationale for a new model before describing the new partnership model and suggest mechanisms derived from the insights of the Capability Approach to strengthen partnerships.

### Why a new model for global academic partnerships?

There is a clear mismatch between what global academic partnerships can achieve and what they are achieving currently. Geopolitical, institutional and personal factors as well as the often short-term, project driven, and inequitable nature of global academic partnerships [4, 8, 14] partly explain the mismatch. Achieving sustainable development through partnerships requires planned, long-term, and equitable relationships between partners. Some funding mechanisms incentivise partnership frameworks with potential for equity [15]. While these are important initiatives, sustainable partnerships must emanate from a sense of institutional freedom. Encouraging partnerships through incentives, legislation or simply from a sense of moral duty is unlikely to work effectively or sustainably [16].

Various frameworks have been put forward to assist with establishing and monitoring partnerships. The ESSENCE research framework [8] sets out seven principles that aim to support the “coordination and harmonization of research capacity investment”. The framework emphasises implementation principles: understanding of local context, local ownership, capacity assessment, research governance, monitoring and sustainability. The Research Fairness Initiative evaluates the fairness of research and partnerships in terms of working along national priorities and equity [11]. Another important principle is set by the Tropical Health and Education Trust (THET), a healthcare delivery partnership [9]. THET sets out eight hallmarks that includes some value propositions, such as reciprocity and respect. It also includes an assessment tool against the eight hallmarks. The Swiss Commission for Research Partnership with Developing Countries [14] offers a guideline with 11 principles, which focus on the planning, implementation and application of research. It also provides questions to explore the nature of the partnership and translation of research findings into societal benefit. Recognising that most of these “north-south” partnership guidelines were developed from the perspective of “northern’ partners perspective, the Canadian Association for Global Health joined up with three “southern” institutions to develop a partnership assessment tool with the objective of developing an agreement on five key elements: sustainability, knowledge production, knowledge translation, capacity development and innovation [10]. The Collaborative Advantage Framework has been developed to maximise the impact of SDG partnerships [17]. The framework describes 10 ways to create additional ‘value’ and maximise advantage for impact and reduce risk. All in all, these are very important frameworks. However, the criticism that they primarily address the perspectives of ‘northern’ institutions and funders is not addressed adequately. Several of the frameworks offer a case for attaining the benefits of partnership rather than proposing mechanisms on building and sustaining partnerships. The values proposed in some of the frameworks are also more business driven than ‘equity’ driven. There is a clear need for a model that suggests a path for establishing a forward-looking partnership anchored in mutual learning, empowerment, autonomy, and freedom.

### Moving towards a mature academic partnership: insights from the capability approach

The Capability Approach emerged as a critique to the ways in which poverty, development and wellbeing were conceptualised and measured [18]. Rather than focusing on resources or income, it argues for freedoms and opportunities, with a focus on capabilities. Development thus conceptualised is about the expansion of freedom and opportunities to be or to do the things individuals have a reason to value [13].

The Capability Approach can offer insights to partnerships in three main areas. First, as a value framework, it brings attention not to mere access to resources but to the freedoms they provide to achieve things of value. Academic partnerships can improve access to resources, infrastructure and skills. However, mature partnerships should reach beyond resources and offer freedoms to enable partners to convert or appropriate resources towards the improvement of well-being or other dimensions they value.

Second, the Capability Approach places attention on diversity among people and their 

circumstances. This implies that their ability to convert resources into capabilities can vary and that evaluations must take context into account [12, 13]. Mature partnerships will require an understanding of the diversity and complexity of circumstances and will explicitly engage with them.

Third, one of the pillars of the Capability Approach is agency and the view of people as change agents [19, 20] with focus on the expansion of agency and the need to address imbalances and power asymmetries that influence it. Mature partnerships should consider all partners to be active agents of change. Alignment of values, and other key elements, such as freedom, trust, and strategic investment are foundational for growing sustainable and mutually beneficial partnerships.

### An academic partnership maturity model

Collaboration maturity models describe the progressive steps that lead to productive relationships between partners for the purposes of pulling strengths together and obtaining competitive advantages. In the context of global academic partnerships, the driving principle needs to be values, particularly in the presence of major cultural chasms and power imbalances.

We propose a values-driven and progressive academic partnership maturity model for global partnerships anchored in equity, mutual benefit, growth, and sustainability. This model was developed through reflexive methods, in which the experience of the authors in participating and supporting partnerships was the main input. The authors collectively have developed more than 30 partnerships in the past two decades.

**Fig. 1:**
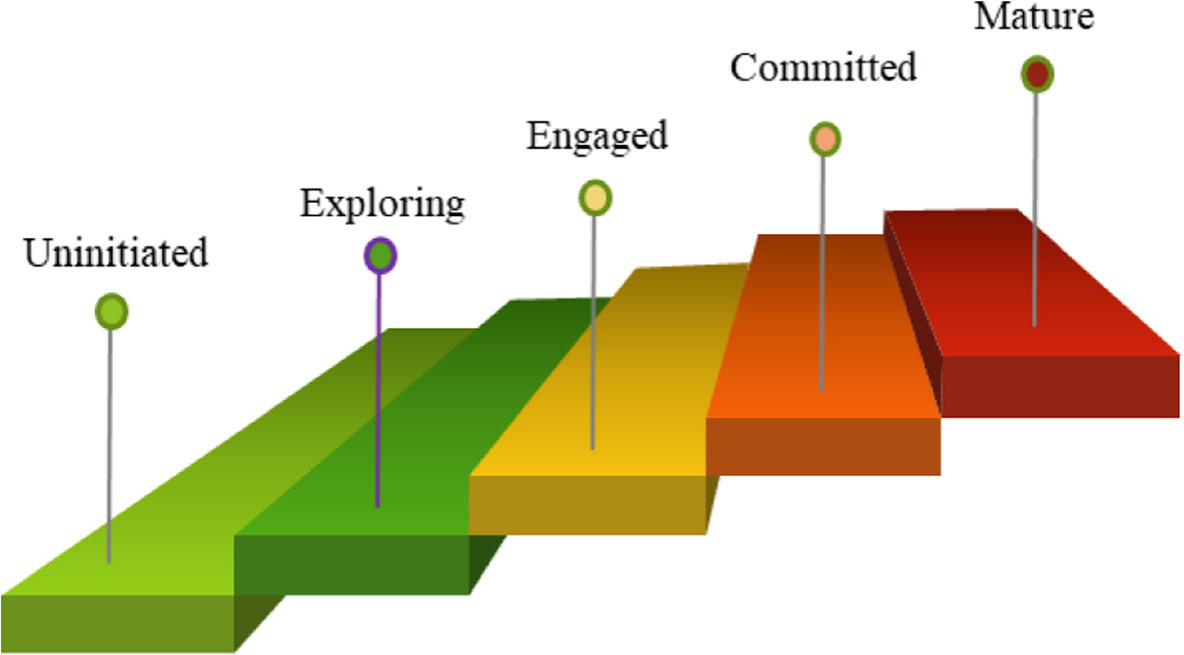
Levels of Academic Partnership Maturity

This model proposes five levels/stages of partnership maturity (Fig. 1). Not all partnerships need to go through all the stages or levels.

#### Level 1: Uninitiated [pre-contemplative]

Institutions at this level of maturity are not interested in or are not aware of global partnerships. These institutions may be helped to contemplate or consider the benefits of engaging in partnership through sensitisation and advocacy. This level is included to ensure that all institutions stand to benefit from what partnerships offer.

#### Level 2: Exploring [Contemplative]

Institutions at this level are interested in the potential of partnerships. They show cultural curiosity and are looking out for or exploring opportunities for partnerships. These institutions may benefit from discussion with institutions with experience as well as organizations that may facilitate partnerships to move to the next stage.

#### Level 3: Engaged

At this level, institutions have established relationships, including engagement in education and/or research. The relationship may take a formalised structure such as a Memorandum of Understanding. They have defined what they and their partners may want. However, these relationships are often unidirectional and superficial, and struggle to take root. Ingressive behaviour may dominate. This is a critical stage in the maturity model where partnerships may die because of discouragement and lack of engagement from senior staff. The institutions must take strategic decisions to give their relationship a chance to grow.

#### Level 4: Committed

There is clear engagement at senior staff level. Firm agreement frameworks are in place and are implemented. Multiple programmes are part of the partnership. Partners have sincere desire and evidence of commitment to each other and to build capacity. Congressive behaviour dominates. Strategic plans that reflect standard partnership capacity building principles are in place.

#### Level 5: Mature

With mature partnerships, formalised relationships beyond individual scientists are in place. Institutional leaders are engaged, and governance and monitoring structures are agreed. The relationship is no longer project specific although multiple projects are common. The relationship is on a sustainable footing. Both parties have confidence in the relationship and benefit from the relationship. There is mutual knowledge and affirmation of culture and deep respect. Joint innovation programmes may take place with the potential for sensitive undertakings, such as commercial exploitation. The partners are committed to strategic investments and there maybe joint infrastructure or other joint centres. The partnership enjoys shared values. Regular evaluations of the relationship at senior management level are important to address any emerging trajectories and engage the relationship in exciting new opportunities.

### Considerations for strengthening partnerships

**Fig. 2:**
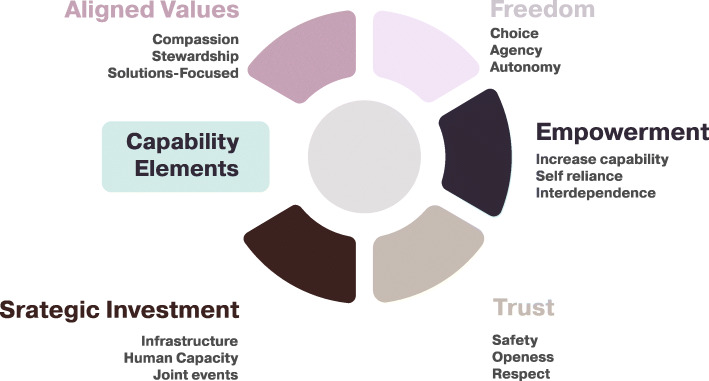
Capability Elements to Strengthen Partnership

Five elements related to the Capability Approach are considered: Aligned Values, Freedom, Empowerment, Trust, and Strategic Investment (Fig. 2).

#### Aligned values

Values that go beyond the usual ‘transparency’ and ‘accountability’ are needed. Compassion, generosity, stewardship, equity, innovativeness and being solutions-focused are values that support reciprocity, dependability, mutual growth, and address any challenges in the relationship or task implementation.

#### Freedom

Is at the heart of the Capability Approach and is related mainly to the ability to have genuine choice. In relation to partnership, this would be related to the ability to choose to be in the partnership and expansion of freedom and the ability to have agency and autonomy. The freedom to move and enjoy and learn from the partnership should be supported. Freedom forms the basis or the foundation of any academic partnership and the prospect of growth and sustainability.

#### Empowerment

Is about enabling partners to grow. It is about creating an enabling space and supplement capability so that the other partner is free to grow, reduce dependence and be self-reliant. This enables partners to contribute more to the partnership.

#### Trust

Is related to safety of operations and relations. How much does one feel safe and confident navigating through a relationship environment. It requires sufficient knowledge of the operation environment, openness, respect, and reciprocity. Sense of safety increases confidence and creates a nurturing environment for the relationship to grow.

#### Strategic investment

This may be considered part of the empowering exchange in the partnership and related to a broader strategic plan. This should include joint investment for creating joint spaces, Research and Development (R&D) infrastructure and expertise as well as opportunities for mutual recognition and affirmation. The African region only accounted for 1.6% of the global investment in R&D in 2016, with marked disparity within Africa [21]. Such strategic investment is fundamental to ensure equitable partnership. This should include joint investment for creating joint spaces, infrastructure as well as opportunities for mutual recognition and affirmation.

We think that increasing incorporation of these elements will lead to stronger and more impactful relationships. These relationships would not be possible without the funders and institutions that support thriving partnerships. As funders are interested in the bigger and longer-term impact of the invested resources, serious considerations should be given by funders to support these elements and offer some provisions for flexibility in the use of funds without compromising accountability.

### The practice aspects of implementing a successful partnership

We attempt to demonstrate the design and implementation aspects of a successful partnership by offering the relationship between Addis Ababa University, Brighton and Sussex Medical School and King’s College London as an example (Boxes 1 and 2). Although there was no deliberate effort, the implementation of this partnership maps well with some aspects of the established practice guidelines, such as the ESSENCE framework [8]. The relationships were established over 15 years and all participants of the partnerships had made a deliberate effort to understand the culture of partner countries and institutions. Decisions around funding allocations, publications and involvement of new partners were made transparently following open discussions. Decisions recognised the relative strengths of each partner. Results, risks, and constraints were reviewed and discussed regularly. Flexibility was one of the hallmarks of the partnership. This was shown particularly during the COVID-19 pandemic when implementation of activities was drastically affected. The flexibility included rescheduling deliverables, and funding reallocations with the consent of the funder(s). As an example, when students were unable to use the cell culture laboratory in the north institution, the decision was made to establish a cell culture laboratory in the south institution and implemented. A new programme of post-doctoral fellowship was developed to ensure sustainable capacity building as well as to build foundations for the next generation relationship. Mobility was also supported to enhance knowledge exchange and equity. Partners worked to negotiate with relevant agencies to ensure that mobility of partners was maintained. There is a need to expand infrastructure and scale up the relationship with more departments within institutions to make the relationship more impactful.

**Box 1:** Design aspects of a partnership

• Beginning of the relationship	
○ Deliberate effort to learn about the culture of partner institutions and countries (Understanding context)	
○ Capacity/resource and needs mapping	
○ Understand power dynamics, which are not always stable	
○ Setting strategic direction for the relationship	
○ Shared inception and agenda	
• Framework of relationship	
○ Transparency regarding allocation of resources	
○ Joint leadership and agreement on coordination	
○ Results-based, anchored in values	
○ Formal institutional agreements	
○ Assess equity over time, including in publications	
• Maintaining relationship	
○ Regular/planned communications (at least monthly scheduled implementation level exchanges) with feedback	
○ Flexibility	
○ Facilitation of mobility	
○ Visiting appointments	
○ Celebration of achievements and accountability	
• Forward looking	
○ Begin from the outset	
○ Focus on both the now and the future (forward looking relationships are more productive in the now and more sustainable)	
○ Review institutional relationship framework and adjust accordingly	
○ Consider longer-term joint institutional relationship with joint ownership	
○ Work on next generation relationships

**Box 2:** Key equity areas

*•* Inception/agenda setting	
*•* Decision making	
*•* Mobility	
*•* Expertise	
*•* Infrastructure	
*•* Publications	
*•* Resource allocation	
*•* Overheads	
*•* Administrative controls	

It is of note that the model is new, untested, and susceptible to the limitations of experiential knowledge. Further development of the framework with additional metrices and assessment tools is needed. The model also does not consider consortia, which may have larger impact with bigger constraints. The current maturity model may be adapted considering the complexities of a consortium.

## Conclusion

We have presented the Academic Partnership Maturity Model with the hope of encouraging at least some global partnerships to enter relationships with clear intent of longer-term value driven engagement. While we recognise that short term, goal driven collaborations are important, we believe that long term partnerships are crucial to attain lasting impact. Such partnerships are more likely to emerge if they are founded on principles of mutual benefit in which experiences and knowledge are exchanged equitably between the partners. Although not highlighted within the model sufficiently, the sustained underinvestment in research infrastructure in academic institutions, particularly in Africa [22], should be considered a major threat to freedom and equitable partnership for development. As long as funders and nations fail to invest in research infrastructure in low-income countries, the freedom that comes through global partnerships will remain a pipe dream.

## Data Availability

Not applicable.
